# Dedicated brain PET system of PET/MR for brain research

**DOI:** 10.1186/2197-7364-2-S1-A63

**Published:** 2015-05-18

**Authors:** Li Cheng, Yaqiang Liu, Tianyu Ma, Shi Wang, Qingyang Wei, Tianpeng Xu

**Affiliations:** Institute of Medical Physics, Department of Engineering Physics, Tsinghua University, Beijing, China

This work is to replace PET ring in human brain PET/MR system with a dedicated wearable PET insert, aimed at improving both patient feasibility and system performance for brain imaging. The designed PET/MR system includes two parts: the inside parts, including a radio frequency (RF) coil and PET ring, are mounted on patient’s head, and the outside part, a MR imager, is dependent of patient. The RF coil is the innermost layer, surrounded by an outer PET-ring layer. They are supported by a MRcompatible structure. And both RF coil and PET detectors are placed inside a standard clinical 3-T MR imager. From the design of the system we can infer that some advantages can be achieved. First, high sensitivity will be achieved with the same amount crystals as the PET ring is more close to region-of-interest area, at a reduced cost. Second, by using a 2-layer depth of interaction (DOI) detector, the parallax effect can be minimized. The resolution will benefit from short positron range caused by magnetic field and smaller ring diameter will also reduce the effect of non-collinearity. Thirdly, as the PET ring is mounted on head, impact of patient motion will be reduced.

